# Describing the roles of civil society organizations in sustaining adolescents’ family planning programs in nine Asian and African countries

**DOI:** 10.1186/s40834-025-00408-w

**Published:** 2025-11-22

**Authors:** Siti Khuzaiyah, Yewo Grace Gondwe, Genuine Agakhan Desireh, Mercy Bolaji, Roseline Florence Gomes, Monir Hossen, Kughong Reuben Chia, Rhino Andrianirina, Fitriyani Fitriyani, Nur Intan Kusuma, Hein Minn Tun

**Affiliations:** 1 Midwifery Program, Faculty of Health Sciences, Universitas Muhammadiyah Pekajangan, Pekalongan, Central Java, Indonesia; 2 Nursing and Midwifery Program, PAPRSB Institute of Health Sciences, Universiti Brunei Darussalam, Bandar Seri Begawan, Brunei Darussalam; 3 Malawi School of Government, Lilongwe, Malawi; 4 Technical Consultant, Daktari Online, Nairobi, Kenya; 5 Technical Support Lead at Stand with a Girl Initiative, Abuja, Nigeria; 6 PAPRSB Institute of Health Sciences and School of Digital Science, Universiti Brunei Darussalam, Bandar Seri Begawan, Brunei; 7 Department of Psychology, Jyoti Nivas College Autonomous, Bengaluru, India; 8 Centre for Advanced Research, Dhaka, Bangladesh; 9 Organization for Health in Sustainable Development, Yaoundé, Cameroon; 10 Catholic University of Madagascar, Antananarivo, Madagascar; 11 Knowledge SUCCESS Baltimore, Baltimore, MD USA; 12 Family Planning, Lilongwe, Malawi; 13 Nasyiatul Aisyiyah, Pekalongan Regency, Central Java, Indonesia

**Keywords:** Adolescent, Family planning, Education programs, Sociocultural engagement

## Abstract

**Introduction:**

Globally, adolescent pregnancy affects around 21 million girls in low- and middle-income countries (LMICs) annually, leading to 12 million births. Family planning education, sexual abstinence, and contraception utilization, mainly for married adolescent girls, are essential to prevent adolescent pregnancy. Although the role of civil society organizations (CSOs) in advancing family planning has been increasingly recognized within the global health, there was lack of systematic documentation of its unique contribution and limited knowledge on its conceptualization and operation on sustainability. This study critically evaluating CSO members in nine carefully selected Asian and African nations involved in Adolescent and Youth Sexual and Reproductive Health (AYSRH) programs, with an emphasis on community involvement, collaboration, and cultural and religious program adaptation.

**Method:**

This critical review study analyzed sustainability factors for adolescent family planning (AFP) programs across nine Asian (Bangladesh, India, Indonesia, Myanmar) and African countries (Cameroon, Kenya, Madagascar, Malawi, Nigeria) through structured review submissions from CSOs practitioners, based on their perspectives. Using thematic analysis, the study identified three key sustainability pillars: (1) community participation, (2) multi-stakeholder partnerships, and (3) cultural-religious adaptation of programs.

**Results:**

This study highlights the crucial role of CSOs in sustaining AFP programs across nine countries in Asia and Africa. Despite an unsupportive situation in the studied countries, such as sociocultural, political, and resource constraints, CSOs provide benefits. Three theme were reflected from the thematic analysis; community engagement and participation (youth empowerment, local ownership, grassroots mobilization, hybrid digital–community models, culturally sensitive advocacy); partnerships and collaboration (government–CSO alliances, joint implementation, cross-sector engagement, data-driven accountability); and cultural and religious adaptation (integrating local values, engaging gatekeepers, addressing barriers through dialogue and innovation).

**Conclusion:**

According to this multi-country study, community engagement, strategic partnerships, and cultural-religious adaptation are the three main pillars that support the effectiveness of family planning programs. The findings of this study advocate for scaled-up collaboration among governments, CSOs, and global health actors to institutionalize contextually grounded solutions. Strengthening CSOs through targeted investments, inclusive program designs, and institutionalizing these strategies within health systems is essential to ensure lasting acceptance, effectiveness, and community ownership.

## Key implications

Civil society organizations (CSOs) play a crucial role in fostering community trust and overcoming societal challenges in sensitive or conflict-affected areas.

Collaboration between CSOs and governments promotes inclusive, adolescent-friendly policies and programs.

Family planning initiatives that align with local religious and cultural beliefs are more accepted and effective.

## Introduction

In 2023, the global adolescent birth rate for females aged 10–14 was 1.5 per 1,000. In LMICs, approximately 21 million adolescent pregnancies occur annually, with half of them being unintended, resulting in 12 million births, of which 50% are unintended [1]. Due to factors like lack of family consent, financial hardships, a long commute to the health center, a lack of decision-making authority, stigma, fear, and exposure to violence, pregnant adolescents may have a low prenatal frequency [2]. Low birth weight, lower activity, pulse, grimace, appearance, and respiration (APGAR) score, preterm birth, stillbirth, premature rupture of the membrane (PROM), maternal anemia, sexually transmitted diseases (STDs), postpartum depression, and maternal deaths are among the medical complications associated with adolescent pregnancies [3, 4]. Preventing adolescent pregnancies and lowering the risk of maternal and neonatal morbidity and mortality are crucial, given the detrimental effects on expectant adolescents and their unborn children^0.1^ Family planning education, sexual abstinence, and contraception utilization, mainly for married adolescent girls, are essential to prevent adolescent pregnancy.

The foundational objective of AFP programs, as articulated by the World Health Organization (WHO), is to cultivate the capacity to ‘avoid, delay, space, or limit childbearing’ through a comprehensive approach [5]. This framework encompasses direct interventions, such as school-based sexual and reproductive health education, and indirect strategies, including conditional cash transfers and economic empowerment initiatives. The global health community has demonstrated increasing commitment to addressing the interrelated challenges of child marriage, adolescent pregnancy, and early childbearing, with CSOs emerging as indispensable actors in operationalizing these commitments across diverse sociocultural contexts [1]. Complementing these efforts, the United Nations Population Fund (UNFPA) advocates for a multisectoral strategy that emphasizes cultivating social assets, particularly peer-based support networks and culturally adapted programming modalities, as essential for enhancing program efficacy and adolescent engagement [5, 6].

The role of CSOs in advancing family planning objectives has been increasingly recognized within the global health discourse. As Boydell et al. (2017) elucidate, these organizations occupy a unique position within the reproductive health landscape, functioning as crucial intermediaries between policy frameworks and community-level implementation [7]. Empirical evidence suggests that CSO-led interventions, particularly those targeting the adolescent population and incorporating social and behavioral change communication components, have demonstrated significant impacts across key indicators, including contraceptive knowledge, method uptake, and client satisfaction. However, the existing literature reveals notable gaps in systematically documenting the specific contributions of civil society actors, as many initiatives employ multi-stakeholder approaches that obscure the distinct inputs of individual partners. At the global level, partnerships such as FP2030 have strategically positioned CSOs as central actors in advocacy coalitions, resource mobilization efforts, and accountability mechanisms [8].

This study addresses critical knowledge gaps by examining the sustainability of AFP programs across nine strategically selected Asian and African countries. The findings are particularly relevant to ongoing efforts to achieve FP2030 commitments, offering evidence-based guidance for optimizing civil society engagement in ways that respect and respond to local sociocultural realities while advancing the fundamental reproductive rights of adolescents.

### Introduction to the national adolescents’ family planning situational update

This section provides a snapshot of the current landscape of AFP across selected countries in Asia and Africa. It highlights the prevalence of adolescent pregnancies, early marriages, access to family planning services, and national policies or programs addressing adolescent sexual and reproductive health. By understanding each country’s unique context, challenges, and efforts, we can identify patterns, gaps, and potential strategies for strengthening AFP interventions globally.

### Indonesia

The National Population and Family Planning Board of Republic Indonesia (*Badan Kependudukan dan Keluarga Berencana Nasional/*BKKBN) reported that 20% of adolescents aged 14–15 and 60% of those aged 16–17 have engaged in premarital sexual activity, decreasing to 20% by ages 19–20 [9]. Additionally, 8% of males and 1% of females reported sexual activity during dating [10]. This behavior contributes significantly to unintended adolescent pregnancies. In Indonesia, the adolescent birth rate in 2021, as reflected in the age-specific fertility rate (ASFR) for females aged 15–19 years, reached 20.49 per 1,000 women of reproductive age (WRA). However, in 2022, the ASFR increased to 26.64 per 1,000 WRA [11], compared with a 2017 birth rate of 24 per 1,000 in urban areas and 51 per 1,000 in rural areas, averaging 36 per 1,000 births [12]. A country with a Muslim-majority population and rich in culture, religion, and culture strongly influences the rule and law in Indonesia [13–15], including the rule of providing family planning for adolescents [16].

### Myanmar

Efforts to tackle inequalities in sexual and reproductive health and rights in Myanmar have been significantly interrupted since the military coup on February 1, 2021, which came after the COVID-19 crisis. Violent repression in response to peaceful demonstrations has resulted in prolonged civil strife that has caused widespread damage to communities nationwide. The crisis has exacerbated the already existing Rohingya refugee situation, as well as a surge in internally displaced populations across the country [17]. Adolescents are at a higher risk, with 16% getting married before turning 18 and fertility rates of 33 per 1,000 women aged 15 to 19 in 2020, indicating a critical gap in access to family planning and reproductive health services, including health education [18, 19]. The dual crisis of political instability and healthcare collapse has created a dire situation, leaving Myanmar’s youth with few resources or support systems to make informed choices about their reproductive health [20].

### India

India is now home to a substantial youth adolescent population, with approximately 253 million youngsters between the ages of 10 and 19 [21]. This integral demographic part witnesses’ significant physiological challenges that are related to sexual and reproductive health, including early pregnancies, concerns with sexually transmitted diseases, and lowered accessibility to contraceptive-based services. These outcomes of early pregnancies result in maternal and child-related health challenges that also perpetuate the cycles of both poverty and lowered learning attainment. Thus, comprehensive family planning educational initiatives are essential in empowering adolescents to make informed choices, strengthen decision-making regarding the delay of early pregnancies, and improve holistic health outcomes [22].

### Bangladesh

By 2030, it is projected that over 35 million adolescents will reside in Bangladesh [23]. The expected population growth in the next few years emphasizes the need to invest in adolescents’ health and well-being. Bangladesh’s government is committed to promoting adolescents’ health and instilling good behaviors in children from a young age. Bangladesh’s Ministry of Health and Family Welfare (MOHFW) is responsible for addressing health issues among adolescents. The MOHFW created the National Strategy for Adolescents’ Health, 2017–2030 [24], to ensure that all Bangladeshi adolescents live healthy, productive lives in safe and supportive environments by 2030. The Ministry of Health and Family Welfare led the effort, alongside UNFPA, UNICEF, WHO, and other development partners, including adolescent groups. The Government of Bangladesh developed the National Adolescents Health Strategy 2017–2030, a strong statement of its commitment to meeting the health needs of this critical population cohort.

### Malawi

Young people in Malawi continue to face significant risks and challenges related to sexual and reproductive health, including unintended pregnancies, child marriages, gender-based violence, and HIV/AIDs [25–27]. Malawi possesses one of the highest rates of child marriage globally, with a prevalence of 37.7% (Multiple Indicator Cluster Survey 2020), despite the 2015 Marriage, Divorce, and Family Relations Act establishing 18 as the minimum legal age for marriage. Poverty, unplanned pregnancies, and ingrained social conventions persist in perpetuating early marriage and adolescent childbirth, which is at 32% (Malawi Demographic Health Survey, 2024) [28]. Over the past years, the government of Malawi has made tremendous strides in addressing major SRH challenges. Malawi has created an enabling environment for addressing youth SRH needs through the adoption and review of policies and legal framework documents such as the Constitution of Malawi (1994) [29], the Gender Equality Act (2013) [30], The Marriage, Divorce and Family Relations Act (2015) [31], HIV&AIDS (Prevention and Management) Act (2018) [32], The Health Act, National SRHR Policy and Strategy [33, 34], Youth Friendly Health Service Strategy (2022–2030) [35], the National Strategy for Ending Child Marriage (2024–2030) [36] among others. Furthermore, Malawi has also been a signatory to international and global commitments that address the reproductive health needs of adolescents and young people. Despite these advancements, there has been slow progress in implementing policy frameworks.

### Kenya

The most recent Kenya National Demographic Survey (2022) revealed that the overall adolescent pregnancy rate has reduced from 18% to 15% [37]. The adolescent pregnancy rate for females aged 15 to 19 in Kenya was 14.8%. As females age, their likelihood of becoming pregnant increases. Consequently, the incidence of adolescent pregnancies was lower among individuals aged 15 at 2.7% in contrast to those aged 19, who exhibited a pregnancy rate of 31.1% [38]. However, the disaggregated picture shows a worrying trend, especially for those who are illiterate or are living in abject poverty. Four in ten adolescent girls without formal education were reported to have been pregnant in comparison to just 5% of young women who had secondary education. Furthermore, young women from the poorest counties are more likely to become pregnant, with some of the arid and semi-arid lands (ASAL) counties recording up to 50% adolescent pregnancy rates. Kenya boasts some of the most progressive policies and guidelines for adolescents and youths in the East African region, with the hallmark guiding documents being the National Guidelines for the Provision of Adolescents and Youth-Friendly Services in Kenya, 2nd edition (2016) and the National Adolescents’ Sexual and Reproductive Health Policy (2015) [39]. Favorable guidelines and policies are also mirrored in other reproductive health policy documents, including the National Reproductive Health Policy, 2022–2032 [40].

### Nigeria

The National Population Commission of Nigeria reports that 23% of girls aged 15–19 have already begun childbearing [41]. Every year in Nigeria, births by adolescent girls ages 15–19 represent half of the 400,000 unplanned births [42]. Results from the 2018 Nigeria Demographic and Health Survey (NDHS) indicate that 19% of adolescents girls aged 15–19 years have started childbearing, and 30% have had a child by the age of 19 [41]. According to the 2023–24 Nigeria Demographic and Health Survey (NDHS), 15% of girls aged 15–19 have begun childbearing—11% have already given birth, 4% are currently pregnant, and 2% have experienced pregnancy loss. This marks a decline from the 2018 NDHS, which reported 19% had started childbearing and 30% had given birth by age 19. Despite the decrease, adolescent pregnancy remains a significant concern, particularly in rural areas and northern regions where cultural norms, poverty, and limited access to youth-friendly reproductive health services persist. These findings underscore the urgent need for community-based, adolescent-responsive family planning programs that prevent early pregnancies, support adolescent mothers, and address regional disparities in access and outcomes. The Nigeria Family Planning Blueprint states that the federal government pays for the purchase of contraceptives through a ‘basket-funding’ mechanism, with external donors, in line with the nation’s policy of providing free contraceptives at all public facilities [43]. It was also found that, in line with this, the government made efforts to increase its annual funding to $4 m for the basket fund, complementing the efforts of external donors to provide free contraceptives for everyone, including adolescents, at all public facilities.

### Madagascar

According to the National Institute of Statistics [44], the proportion of the Malagasy population under 15 years of age represented 41.3% of the total population, while those under 30 years of age represented 70.6% or 18,130,813 individuals in 2018, thus exposing Madagascar as an island with an extremely young population. Hence, this situation highlights the importance of young adolescents to the Malagasy population Faced with impulsive youth, the lack of leisure and money generates harmful effects for them, such as early pregnancy. [45]. As a result, these juvenile problems are part of the government’s constant concerns. Although the rate of contraceptive use is high in Madagascar, the birth rate is high, too, leading to an increase in the infant mortality rate [46]. This contradictory reality is because few Malagasy people, including young people, use contraception for fear of being judged by society as sexually ill [47].

### Cameroon

Cameroon’s population is predominantly young, with more than half of the population in 2019 being under 20 years of age, while those under 15 years old represent 42.5% of the total [48]. According to the third Demographic and Health Survey, the age of first sexual intercourse in Cameroon in 2004 was 16.5 years [49]. This age has gradually decreased over the years, due in part to modernization and the advent of social media. According to Foumane et al., the average age of sex debut in Cameroon was 15.3 years, and 21.3% of adolescents who were schooling in the urban city of Yaoundé were sexually active [50]. Another report by MICS in 2014 showed that 16.0% of young women and 9.3% of young men aged 15 - 24 years in Cameroon had sex before reaching 15 years of age [51]. This early sexual debut in adolescents who are inexperienced in sexuality and sexual and reproductive health usually predisposes these adolescents to adverse consequences. In 2011, 17.9% of adolescents aged 15 - 24 years reported having had an STI [52]. A 2014 report in Cameroon showed that 27.5% of women aged 20 - 24 years had at least one live birth before their 18^th^ birthday [53].

## Method

Using a qualitative critical review methodology and purposeful sampling, the study selected one representative from nine countries: Indonesia, Myanmar, India, Bangladesh, Malawi, Kenya, Nigeria, Madagascar, and Cameroon. These individuals were chosen to provide practical, on-the-ground insights of CSO practice on implementation and sustaining Adolescent and Youth Sexual and Reproductive Health programs.

Using a qualitative critical review methodology, this study drew directly on the viewpoints and experiences of CSOs practitioners in nine Asian and African nations. Indonesia, Myanmar, India, Bangladesh, Malawi, Kenya, Nigeria, Madagascar, and Cameroon were among the nations included in the review. The nine nations were chosen with strategy and intent. The active participation of practitioners from CSOs that are part of the Community of Practice on Adolescent and Youth Sexual and Reproductive Health (CoP-AYSRH) led to the selection of these nations. Due to their proven track record and active participation in teenage family planning initiatives within their respective national settings, contributors from these nations were selected. In addition to contributing context-specific expertise from various sociocultural and geographic regions in Asia and Africa, their involvement in this global community of practice guaranteed a shared awareness of AYSRH priorities. Voices that are both well-versed in global reproductive health discourses and have a strong foundation in grassroots implementation were included, thanks to our purposeful sample technique. Their proficiency ensured the calibre, legitimacy, and depth of the critical thoughts presented in this research.

Selected CSO representatives from each participating nation were given access to an editable and organized Google Document that the primary author produced to gather data. Every contributor was instructed to record their observations in a section designated for their respective nation. The following instructions were given to the contributors to guarantee uniformity and pertinence among submissions:

- Provide a concise summary of their nation’s current adolescent pregnancy problem.
- Explain how family planning use is influenced by social, economic, religious, and belief systems, paying particular emphasis to adolescents.
- Describe the family planning education programs being offered in their nation, including the role of NGOs and CSOs in these programs.
- Provide a critical analysis of their experiences and viewpoints on maintaining AFP programs in their nations. The three main sustainability pillars that were the focus of the critical assessment were: 1) community involvement, 2) multi-stakeholder partnerships, and 3) program modification for cultural and religious reasons.

The compiled narratives were thematically analyzed to identify common and divergent factors influencing the sustainability of AFP programs in different contexts. To determine the common and unique aspects affecting the viability of adolescents’ family planning programs across various contexts, the collected narratives were subjected to a thematic analysis.

### Data analysis

The analytical process employed thematic analysis to identify cross-cutting patterns in program sustainability across the nine implementation contexts. Instead of providing a comparison analysis, a theme synthesis was conducted as part of a qualitative critical assessment based on the data gathered from each country’s experience, aiming to identify recurrent patterns, methods, and contextual adjustments. The study yielded three themes: (1) Cultural and Religious Adaptation; (2) Partnerships and Collaboration; and (3) Community Engagement and Participation. These themes, which represent both contemporary practices and aspirational methods meant to ensure program continuity, arose from CSO-reported experiences and thoughts. The data do not provide conclusive evidence of long-term sustainability following external support, despite some examples such as community ownership or integration into government institutions, demonstrating partial or ongoing sustainability. Limited data on program continuation and financing sources make it difficult to evaluate sustainability outcomes comprehensively. From the standpoint of CSOs actively involved in adolescent and AYSRH activities, the theme synthesis instead provides a framework based on perceived facilitators of sustainability.

### Result

### Theme 1: CSO roles in community engagement & participation for sustaining AFP programs

#### A) Peer education & youth empowerment

CSOs actively promote peer-led education as a culturally sensitive and effective method to reach adolescents with accurate and relatable information on sexual and reproductive health (SRH). *The Rashtriya Kishor Swasthya Karyakram* (RKSK), India’s National Adolescents’ Health Program, incorporates dynamic approaches that encompass peer educational models, school-driven programs, community outreach, and digital forums to achieve accessibility. In Madagascar, CSOs regularly organize awareness campaigns in schools and local communities to disseminate crucial reproductive health information [54, 55]. In Nigeria & Malawi, CSOs have capacitated youth community-based distribution agents who have supported the delivery of family planning and SRH services. Over time, this has bridged the access gap to services among young people living in hard-to-reach areas. Young people have also been trained on SRHR advocacy and social accountability [56].

In Bangladesh, CSO-developed program activities based on contraceptive access issues like stigma, misleading information, and provider bias, adolescent peer networks and youth groups developed contextually relevant remedies [57, 58]. Young-led conversations generated tools for local youths as well as outreach materials.

In Cameron, the Centre for Community Regeneration and Development (CCREAD) addresses persistent misconceptions and cultural stigma surrounding modern contraceptives through peer education networks, intergenerational dialogues, and rural outreach, with a focus on male involvement, spousal communication, and family wellbeing [59]. CCREAD also incorporates family planning awareness into vocational training institutes and community learning centres as part of its Education for Sustainable Development framework, thereby guaranteeing that disadvantaged youth and women not only acquire livelihood skills but also accurate knowledge of reproductive health and contraception [60].

A CSO in Indonesia offers a comprehensive health services program for adolescents, called *Pelayanan Remaja Sehat Milik Nasyiatul Aisyiyah* (PASHMINA) in Bahasa. Adolescents have expressed satisfaction with the program’s reproductive health counseling and six service stations [61]. Additionally, preventive measures aimed at addressing adolescents’ reproductive health issues, especially within schools, have been implemented through initiatives like the Adolescent-Friendly Health Services (called *Pelayanan Kesehatan Peduli Remaja, abbreviated as PKPR*) [62] and the Planned Generation Program (called *Generasi Berencana*, abbreviated as GenRe) [63].

#### B) Local ownership, grassroots mobilization & gatekeeper engagement

CSOs build trust and mobilize local communities, including parents, teachers, traditional rulers, and religious leaders, to normalize family planning discussions and services. In Nigeria, for example, Intergenerational dialogues are held with parents, traditional rulers, and pharmacists. In Ebonyi State, community-embedded ASRH interventions saw success through stakeholder consultations, aligning services with cultural norms, and improving adolescent access.

In Bangladesh, CSOs have encouraged community involvement for local ownership, trust-building and grassroots participation especially with teenagers in the designing and sustainability of the AFP program [64, 65]. Among the gatekeepers, CSOs arranged meticulous visits and community forums for religious leaders, parents, teachers, and local government officials in order to enable youth from rural and semi-urban communities to access AYSRHR service such as teenage contraception and participatory planning sessions to alter perceptions [66, 67].

In India, the Society for Nutrition, Education & Health Action (SNEHA) initiated a community mobilization initiative in Mumbai to improve sexual and reproductive health outcomes. Through direct engagement with community members, they facilitated open discussions, addressed social taboos, and provided essential education about contraception and STI prevention [68]. Additionally, CSOs-led programs employ various strategies, including peer education models, where trained adolescents facilitate discussions. This has been integral to the RKSK, India’s National Adolescents Health Program [69]. Furthermore, School-based programs, such as the adolescents’ needs Udaan initiative in Jharkhand [70] and programs in Odisha [71] significantly improved knowledge and attitudes toward family planning among female adolescents.

In Cameroon, community engagement and participation are pivotal in advancing family planning (FP). A 2023 pilot in urban slums by Cameroon Baptist Convention Health Services trained community health workers (CHWs) to deliver FP services, significantly reducing unintended pregnancies and illustrating how local capacity‑building yields strong results [54]. Effective CSO and CHW involvement elevates trust, improves access, and bridges resource gaps. Digital platforms like BornFyne-PNMS, developed through participatory stakeholder processes, show how community voices enhance design and uptake. Collaborative consultations with CSOs, women, men, and CHWs revealed critical cultural and informational barriers, such as concerns about side effects and spousal communication, which informed tailored messaging aligned with the Health Belief Model [55].

#### C) Digital and community-based hybrid models

CSOs leverage digital platforms alongside in-person engagement to expand their reach, particularly in marginalized or urban areas with limited internet access. In Myanmar, for example, Digital tools like the *Ma Shet Ne* campaign and the *Baykin 2 app* deliver SRH education. With the support of Global Affairs Canada, UNFPA Myanmar has launched the Baykin 2 mobile application, partnering with the 360ed team. Integrating with Augmented Reality (AR), the Baykin 2 app is designed to introduce adolescents and young people in Myanmar to key topics on sexual and reproductive health & rights, gender equality, gender-based violence, and other youth-related content, such as self-defense. The Baykin 2 app integrates learning and gaming to increase the engagement of young users and provide better visuals for learning about their bodies and rights. It additionally seeks to equip adolescents and young people with knowledge and tools to be aware of and thus less vulnerable to gender-based violence [72].

In Bangladesh, participatory planning and youth-generated materials are supported through digital access. The digital health landscape in Bangladesh has been flourishing in recent years, with an influx of e-health, m-health, telemedicine, and new mobile apps. These services include online consultations with doctors, at-home medicine delivery, and other diagnostic solutions. It’s available digital health services, with a special focus on mobile apps that address SRHR [73].

In Cameroon, *the BornFyne app, co-designed with community input,* improves uptake and relevance. The BornFyne project’s goal is to achieve optimal health across the lifespan of the population it serves through the use of advanced digital health systems. To improve maternal and child health, the project focuses on providing quality antenatal care, ensuring prompt reporting, utilizing high-quality data, and leveraging e-networking. By doing this, the project, in collaboration with the Department of Family Health, is making a step toward the achievement of the sustainable development goals and the country’s goal of 2035. The research result underscores the relevance of engaging a diverse group of stakeholders as a strength in intersectoral collaboration and partnership in implementing digital health interventions. It ensures that the views and experiences of those directly impacted by the intervention are considered, and it contributes to a more well-rounded and impactful assessment of the BornFyne-PNMS platform’s role in improving RMNCAH (Reproductive, Maternal, Newborn, Child and Adolescent Health) in rural settings [74].

#### D) Contextual & cultural sensitivity in program design

Programs are locally adapted to reflect social, cultural, and religious contexts for greater acceptability and effectiveness. For example, as a nation with a Muslim majority, Indonesia is deeply influenced by its rich cultural, religious, and customary practices, which significantly affect the rules and laws governing various aspects of life, including family planning for adolescents [14, 15, 18]. This cultural framework plays a crucial role in shaping the regulations surrounding providing family planning services to adolescents [16]. Encouraging and offering contraception to married adolescents is acceptable and practicable. However, offering it for unmarried adolescents appears to be an impossible task, as it is viewed as controversial by society due to moral and religious concerns, conflicts with social and cultural norms, andstigma regarding adolescents’ sexuality. Contraceptive services are restricted to married adolescents to adhere to *Sharia* principles, which prohibit altering the status of *halal* to *haram* or vice versa. For unmarried adolescents, sexual abstinence is encouraged to prevent adolescent pregnancy.

In Nigeria, cultural and religious adaptation has been a critical strategy employed by CSOs in sustaining AFP programs. Recognizing the influence of cultural norms and religious beliefs on adolescent sexual and reproductive health, CSOs have worked closely with community gatekeepers including religious leaders, traditional rulers, and parents to foster acceptance and reduce resistance to family planning messages. CSOs have worked closely with community gatekeepers, including religious leaders, traditional rulers, and parents, to improve acceptance of family planning messages. By framing family planning within culturally acceptable narratives, such as child spacing for maternal and child health or fulfilling religious duties of safeguarding life, CSOs have been able to engage conservative communities more effectively. In regions with high rates of early marriage, mainly in rural areas, interventions have integrated faith-based messaging and partnered with religious activist to provide accurate information and motivaitons.

Kenya is a deeply culturally and religiously diverse country with different tribes, regions, and religions holding their own views on adolescent and reproductive health rights and the implementation of such programs. Strong traditional political influences, male hegemony, and religious ideologies have been the main barriers to progressive national policies since the early 2000s [75]. Programmes by CSOs have found innovative ways to tackle these cultural and religious barriers by first ensuring they understand the cultural, social, and economic contexts of the areas they are working in, then tailoring their approach and messages to align with these contexts. Other CSOs have made use of male champions to promote the active involvement of men in reproductive health, and a consortium of Kenyan religious leaders has joined forces to combat adolescent pregnancies and gender based violence [76].

#### E) Advocacy, social accountability & policy influence

CSOs strengthen community voice and advocacy capacity, ensuring youth and community stakeholders participate in program design, evaluation, and policy dialogue. In Kenya, for example, the government, through the Ministry of Health, has established a reproductive health technical working group that meets quarterly to track implementation progress, synergize approaches, and commit resources to priority SRH areas outlined in the government’s annual work plan. This collaborative approach ensures that CSOs contribute to the most pressing issues at hand while also highlighting areas that may not be receiving adequate attention and resources. Recently, in response to utterances undermining the right to access education by pregnant learners, CSOs put out a joint statement firmly opposing the statements made by politicians and reaffirming that access to education is a critical component of ensuring adolescent sexual and reproductive health rights (ASRHR) are upheld [77].

In Bangladesh, young people participate in policy dialogues and hold service providers accountable. Recent research conducted in Bangladesh suggests that group decision-making, inclusive dialogue, and participatory planning can help increase the relevance, ownership, and sustainability of AFP projects [64, 67]. Communities and service networks were part of CSO to guarantee that the efforts reflected the realities and needs of adolescents in diverse contexts.

In Nigeria, key strategies include training youth ambassadors, integrating pharmacists and patent medicine vendors into FP delivery, and organizing intergenerational dialogues with parents, religious leaders, and traditional rulers. Additionally, the Challenge Initiative (TCI) supports local governments and CSOs in using data to strengthen community accountability and tailor services [78]. The summary of CSOs’ roles in community engagement & participation for AFP sustainability is available in Table 1

**Table 1:** Summary table of the role of CSOs in community engagement & participation for AFP sustainability

Thematic Role	Country Example	Key CSO Activities
Peer Education & Youth Empowerment	Cameroon, Bangladesh, Indonesia, India, Madagascar, Malawi, Nigeria	Peer-led sessions, youth ambassadors, training of adolescent educators, and comprehensive health service program for adolescents (PASHMINA, PKPR, GenRe).
Local Ownership, Grassroots Mobilization & Gatekeeper Engagement	Bangladesh, Cameroon, India, Nigeria	Community forums, intergenerational dialogue, religious/traditional leader involvement, SNEHA, participatory engagement in designing digital platform, and collaborative consultation.
Digital & Community Hybrid Models	Bangladesh, Cameroon, Myanmar	Digital campaigns (Ma Shet Ne), youth-designed materials, online SRHR platforms (Baykin 2 app, BornFyne app).
Contextual & Cultural Sensitivity in Program Design	Indonesia, Kenya, Nigeria	Sharia-sensitive approach, cultural adaptation strategy, work with community gatekeepers, male champion and involvment for AFP
Advocacy, Social Accountability & Policy Influence	Bangladesh, Kenya, Nigeria	Health technical working group, joint statement ensuring ASRHR, youth ambassador training, patent and pharmacist integration, intergenerational dialogue, The Challenge Initiative (TCI)

### Theme 2: CSO roles in sustaining AFP under the theme of partnerships & collaboration

#### A) Policy advocacy through government-CSO alliances

In many countries, CSOs act as policy partners, helping shape and implement inclusive reproductive health policies by collaborating with ministries, technical working groups, and interagency coalitions. In Nigeria, for example, CSOs partner with ministries and contribute to national platforms such as FP2030 and Track20 [8, 79]. The Nigeria Family Planning 2030 (FP2030) commitments have encouraged collaboration, with CSOs contributing to accountability mechanisms such as the Family Planning Dashboard and Track20 data systems [80].

In India, CSOs support government plans, such as FP2020/FP2030 and the RMNCH+A framework, through long-term collaboration. India’s vision for sustaining family planning programs is founded on a rights-based, inclusive, and holistic approach that is consistent with the overarching aims of universal health care and sustainable development. The government prioritises voluntary access to contraceptive options, excellent service delivery, and informed decision-making, guided by national plans such as the National Population Policy (2000) and initiatives like Family Planning 2020 (FP2020) and, more recently, FP30. The government aims to enhance health systems by integrating family planning into the Reproductive, Maternal, Newborn, Child, and Adolescent Health (RMNCH+A) framework, with a focus on equity, particularly in underserved areas. To ensure continuity and scale, we prioritize long-term investments in community outreach, supply chain management, capacity building, and demand generation. India also recognizes the need to engage adolescents and young people, encourage male participation, and harness digital health advances to increase reach and responsiveness.

In Malawi & Kenya, CSOs join technical working groups (TWGs) to ensure youth needs are reflected in national policies. In Malawi, the government has established technical working groups that jointly mobilize CSOs and key government stakeholders to plan, implement, and monitor SRHR interventions. The Youth Friendly Health Services TWG has collaborated to ensure that policies reflect the needs of young people and improve service delivery, particularly for young people living in rural settings, key populations, and persons with disabilities [81]. Civil society networks at the district and national levels have also supported coordination, technical expertise, and resource mobilization for SRHR interventions. While in Kenya, the government, through the Ministry of Health, has established a reproductive health technical working group that meets quarterly to track implementation progress, synergize approaches, and commit resources to priority SRH areas outlined in the government’s annual work plan. In addition to this technical working group, CSOs have openly collaborated with key stakeholders in the reproductive health space, including county departments of health, reproductive health coordinators, and grassroots youth-led organizations, to deliver and scale up youth-friendly sexual and reproductive health services [77].

#### B) Joint program implementation and service delivery

CSOs co-deliver adolescent-friendly services with governments and international agencies, bridging gaps in access, infrastructure, and local trust. For example, in Indonesia, the government’s *Posyandu* initiative integrates services with support from peer groups and CSOs. The Indonesian Ministry of Health has launched various health education initiatives aimed at adolescents, focusing on reproductive organ care, puberty development, the effects of pornography, unintended pregnancies, HIV/AIDS, and the importance of delaying marriage. These efforts involve collaboration among the government, parents, and peer groups [82]. A key service for adolescents’ reproductive health education is the Integrated Health Service (called *Pusat Pelayanan Terpadu*, abbreviated as *Posyandu*), which was guided by government regulations established in 2018. The Posyandu includes five service stations and has recently been integrated into Village Community Institutions (*Lembaga Kemasyarakatan Desa* or *LKD*) to provide comprehensive services across all life stages [83].

In Bangladesh, BRAC partners with the government and global institutions to co-develop culturally grounded programs. Bangladesh Rural Advancement Committee (BRAC) “Adolescents Development Program” in Bangladesh effectively integrates family planning messages within local cultural and religious norms. By working alongside government institutions and international organizations, BRAC ensures its programs are culturally sensitive and widely accepted [84]. Save the Children’s Adolescents Development Program also highlights the importance of family involvement in discussing sensitive issues such as menstruation, contraception, and early marriage, ensuring that these topics are addressed in a supportive environment [85]. Moreover, participatory education models, such as peer-led education projects, have proven effective in empowering adolescents to become advocates for their reproductive health.

In Cameroon & Madagascar, CSOs collaborate with national and international bodies, such as UNFPA and FID, for outreach and service provision. In Cameroon, the government has demonstrated a strong commitment to enhancing the health of its citizens through a health sector strategy aimed at reducing unmet family planning needs, particularly among adolescents, by at least 25% by 2027. To achieve this objective, the government has collaborated with UNFPA and other development partners to support initiatives that empower women, especially adolescent girls [86], in improving their access to family planning services. This initiative aims to achieve the dual goal of addressing unmet family planning needs and promoting reproductive health. The Malagasy government, concerned about the future of Malagasy youth, has strengthened its collaboration with CSOs in the field of family planning. This collaboration encompasses joint initiatives between the two parties aimed at enhancing access to reproductive health services. In March 2022, for example, Marie Stopes Madagascar signed a partnership agreement with the Development Intervention Fund (FID) to raise awareness of family planning among certain households and provide them with appropriate services [87]. In addition, the Fitia Association, chaired by the First Lady of the Malagasy people, has extended its partnership with UNFPA until December 2023, with the aim of strengthening actions to combat gender-based violence and promote family planning [88]. These collaborations strengthen the commitment of both parties Malagasy government and CSOs to improving reproductive health and promoting the rights of women and girls in Madagascar.

#### C) Multi-stakeholder and Cross-sector partnerships

CSOs often facilitate multisectoral collaboration, linking health, education, religious, and youth-led groups to maximize program sustainability. In Nigeria, for instance, alliances are being formed with youth-led organizations, healthcare providers, and traditional leaders through initiatives like The Challenge Initiative (TCI) [78]. The CSOs have forged strong alliances with government ministries, donor agencies, youth-led groups, traditional leaders, and healthcare providers to scale up access to adolescent-friendly services. These partnerships have enabled coordinated efforts in policy advocacy, service delivery, and community engagement. For example, through TCI, CSOs and local governments co-implement AYSRH programs using a coaching model that empowers local actors to own and sustain interventions.

In Kenya, CSOs collaborate with county health departments, reproductive health coordinators, and grassroots organizations. CSOs have openly collaborated with key stakeholders in the reproductive health space, including county departments of health, reproductive health coordinators, and grassroots youth-led organizations, to deliver and scale up youth-friendly sexual and reproductive health services [77].

In Cameron, CCREAD has integrated AYSRH education into its broader community development and sustainability programs, thereby complementing national and CSO-led family planning initiatives [89, 90]. In addition, it collaborates with municipal health facilities and authorities in the South-West Region to increase the availability of services that are appropriate for adolescents [91, 92]. CCREAD illustrates how integrated community regeneration models can improve the sustainability and acceptability of AFP programs by connecting family planning education to environmental stewardship, women’s empowerment, and livelihoods [90].

In India, partnerships span across civil society, the private sector, and multilateral development partners. To assure continuity and scale, we prioritize long-term investments in community outreach, supply chain management, capacity building, and demand generation. India also recognizes the need to engage adolescents and young people, encourage male participation, and harness digital health advances to increase reach and responsiveness. Collaborations with civil society, the private sector, and multilateral partners are crucial to advancing this strategy, ensuring that family planning remains a cornerstone of the country’s public health and socioeconomic development.

#### D) Strengthening accountability and data-driven planning

CSOs contribute to accountability mechanisms and use data for improved planning, transparency, and responsiveness in AFP programs. For Example, in Nigeria, they participated in Track20, FP dashboards, and data sharing to enhance impact monitoring. Track20 has focused on strengthening the use of country-generated family planning (FP) service statistics by improving data quality and promoting their strategic use for monitoring progress and decision-making [79]. To ensure sustainability, Track20 integrates data review and analysis functions directly into national Health Management Information Systems (HMIS), primarily using the DHIS2 platform. One of its key contributions is the development of a Family Planning DHIS2 Module, which provides policymakers with dashboards, tools for program analysis, data quality checks, and an automatic calculation of the Estimated Modern Use (EMU) indicator for monthly monitoring. Several countries, including Nigeria, have adopted this module. To make these tools more widely accessible, Track20 launched FP DataPro, the first DHIS2 app dedicated to family planning, which is available through the DHIS2 App Hub. FP DataPro offers the same features as the FP module and has been expanded to include a Maternal and Newborn Health (MNH) module. It comes with administrator and user manuals to help countries customize and implement the app according to their needs. Some countries, including Nigeria, have transitioned from the original FP module to the enhanced FP DataPro App, improving efficiency and enabling more robust data use in family planning programs [79, 93, 94].

In Kenya, quarterly Technical Working Group (TWG) meetings, convened with active participation from CSOs, serve as a vital forum for identifying service delivery gaps in family planning and setting strategic priorities. These meetings provide a platform for sharing data and discussing challenges, such as contraceptive stockouts, logistics bottlenecks, and training needs, at both district and facility levels. For instance, a TWG meeting on May 8, 2012, addressed the understock of injectable contraceptives (e.g., DMPA) and condoms, and explored options for improving supply chain management, including enhancing commodity flow through KEMSA and strengthening district store systems. Through this collaborative, data-driven approach, CSOs contribute local insights, ensure accountability, and help guide evidence-based decisions to improve the quality and efficiency of FP service delivery across Kenya.

The summary of CSO roles in sustaining AFP under the theme of partnerships & collaboration is available in Table 2.

**Table 2:** The summary table of CSO roles in sustaining AFP under the theme of partnerships & collaboration

Thematic Role	Country Example	Key CSO Activities
Policy Advocacy through Government-CSO Alliances	India, Kenya, Malawi, Nigeria	Partner with ministries and national platform such as FP2030 and Track20 data system, FP Dashboard, RMNCH+A framework, joint TWGs
Joint Program Implementation and Service Delivery	Bangladesh, Cameroon, Indonesia, Madagascar	Posyandu, BRAC, Marie Stopes Madagascar and Fitia Association partnership with UNFPA.
Multi-Stakeholder and Cross-Sector Partnerships	Cameroon, India, Kenya, Nigeria	Multi stake holder alliance (TCI), empowers local actors, CCREAD, collaborate with health department, health coordinators, grassroots organization, other CSOs, and private sector.
Strengthening Accountability and Data-Driven Planning	Kenya, Nigeria	Use of tools like FP2030 dashboards, Track20, FPData Pro App, quarterly TWG reviews, supply chain management.

### Theme 3: CSO role in cultural and religious adaptation for sustaining family planning programs

#### A) Integrating local cultural and religious values

CSOs have strategically embedded family planning messaging within local cultural and religious frameworks to increase community acceptance and reduce resistance. In Bangladesh, BRAC and Marie Stopes incorporate cultural and religious norms into programs to normalize family planning. In Bangladesh, BRACs Adolescents Development Program and Marie Stopes Bangladesh have successfully incorporated local cultural and religious values into their messaging, making discussions on family planning more socially acceptable [95, 96].

In Nigeria, CSOs engage religious leaders and traditional rulers, framing family planning in terms of child spacing and religious duties. Cultural and religious adaptation has been a critical strategy employed by CSOs in sustaining AFP programs in Nigeria. Recognizing the influence of cultural norms and religious beliefs on adolescent sexual and reproductive health, CSOs have worked closely with community gatekeepers—including religious leaders, traditional rulers, and parents—to foster acceptance and reduce resistance to family planning messages [97]. By framing family planning within culturally acceptable narratives, such as child spacing for maternal and child health or fulfilling religious duties of safeguarding life, CSOs have been able to engage conservative communities more effectively. In regions with high rates of early marriage, interventions have integrated faith-based messaging and partnered with religious institutions to provide accurate information and referrals. These culturally sensitive approaches have increased community trust, reduced stigma around contraceptive use, and opened dialogue between adolescents and influential adults, making AFP programs more sustainable and locally owned [98].

In Cameroon, programs like BornFyne adapt messaging to local dialects and respect religious norms, promoting acceptance of modern contraception. Cameroon’s diverse religious landscape—comprising 66% Christian, ~30% Muslim, and traditional faiths—deeply influences family planning (FP) uptake in the country [99]. In some rural Muslim and Christian communities, religious doctrine frames modern contraception as sinful, making natural methods or abstinence more culturally acceptable. For example, in Bamenda, Catholic church settings promote natural family planning, with men often dominating FP decisions [100]. Cultural factors valuing large families for economic security and high child mortality cause resistance to limiting births [101]. However, FP programs like BornFyne adapt messaging by using local dialects, addressing side-effect fears, integrating male engagement, and respecting religious norms, improving acceptance [74]. Moreover, CCREAD guarantees that family planning initiatives are congruent with the religious and cultural contexts of the local area by customising its approaches [91, 92]. This approach enhances program acceptance and mitigates resistance that is based on cultural or religious beliefs.

#### B) Engaging community gatekeepers and families

CSOs collaborate with families, religious leaders, and community gatekeepers to create supportive environments and promote open dialogue on sensitive topics. In Kenya, CSOs involve male champions and religious leaders to challenge gender norms and reduce adolescent pregnancies. Kenya is a deeply cultural and religious country with different tribes, regions, and religions holding their own view on adolescent and reproductive health rights and the implementation of such programmes. Strong traditional political influences, male hegemony, and religious ideologies have been the main barriers to progressive national policies since the early 2000s [102]. However, the revised 2010 constitution is credited for breaking the strong religious and cultural narratives, allowing for more CSOs to engage in AYSRH in the country and increased collaboration between the government and CSOs as initiatives that employed a multi-sectoral approach were scaled nationwide, showing promising signs of sustainability through the involvement of relevant ministries [75]. Programmes by CSOs have found innovative ways to tackle these cultural and religious barriers by first ensuring they understand the cultural, social, and economic contexts of the areas they are working in, then tailoring their approach and messages to align with these contexts. Other CSOs have made use of male champions to promote the active involvement of men in reproductive health, and a consortium of Kenyan religious leaders has joined forces to combat adolescent pregnancies and gender based violence [76]. Ultimately, these approaches reflect the need for a multi-sectoral approach to tackling SRH issues. In Bangladesh, Save the Children considers family involvement to be a key to discussions on early marriage and contraception, promoting adolescent support. In India, SNEHA engages community leaders and parents to overcome taboos and encourage supportive adolescent environments.

In Cameroon, CCREAD fosters intergenerational dialogue and confronts obstacles that are founded on stigma and falsehoods by engaging families and community influencers. These endeavours reinforce the notion that family wellbeing is essential for the reproductive health of adolescents by promoting male participation and interpersonal communication [59].

#### C) Utilizing innovative and interactive approaches

Interactive, culturally sensitive methods are employed to break taboos and encourage informed decision-making among adolescents. In Bangladesh, theatre, storytelling, and games are used for engagement. Save the Children’s initiatives have involved families in discussions about early marriage and contraception, ensuring that adolescents receive support from their immediate social environment [88]. People have also used interactive methods such as theater, storytelling, and games to engage with sensitive topics. These methods have proven highly effective in encouraging behavior change and dismantling taboos. Workshops promote critical thinking and problem-solving in the context of reproductive health. Pathfinder International’s programs have utilized interactive workshops to educate adolescents about reproductive health and family planning, while fostering critical thinking and problem-solving skills [103].

In Madagascar, peer educators and community workers raise awareness to counter stigma and myths. In Madagascar, few Malagasy people, including young people, use contraception for fear of being judged by society as sexually ill. [47]. Sexuality is still a taboo subject in Malagasy society [104]. To protect these women and support young adolescents, who are vulnerable to social pressures, a lack of sex education, and myths surrounding contraception, many actions have been carried out in the different regions of the island, such as the establishment of health centers, but also awareness-raising among young people by community workers or young peer educators on sexual health [105].

#### D) Addressing sociocultural barriers through dialogue and transformation

CSOs utilize transformative dialogues and values clarification to alter attitudes, dismantle taboos, and foster gender equity. In Malawi, gender-transformative approaches and intergenerational dialogues are shifting attitudes toward SRHR acceptance to address rigid gender norms and existing power dynamics. CSOs have utilized values clarification and attitude transformation exercises to change negative attitudes and misconceptions surrounding adolescents’ access to SRHR. Additionally, transformative intergenerational and peer-to-peer dialogues have contributed to dismantling taboos and promoting the acceptance of SRHR education in communities [9].

In India & Indonesia, CSOs address deep-rooted norms and restrictive policies by collaborating with healthcare workers and integrating local values. In India, CSOs encounter several challenges when implementing family planning education programs. Despite their essential roles, various concerns hinder the effective delivery of family planning education to adolescents. Deep-rooted sociocultural norms and taboos surrounding discussions of sexuality create significant obstacles. Caregivers and community leaders often view this type of education as irrelevant, which can lead to resistance that negatively impacts program implementation [106]. To address these challenges, CSOs collaborate with community members, parents, and healthcare workers to foster supportive environments for adolescents. For instance, in Mumbai, SNEHA mobilize community to improve sexual and reproductive health results [68].

In Indonesia, a nation rich in culture, preventing adolescent pregnancy is a complex task that encompasses not just adolescents but also the wider society and sociocultural background. There are approximately 618,000 CSOs in Indonesia, consisting of both religion-affiliated and non-religion-affiliated organizations [107]. Among them, at least ten are Islamic-affiliated CSOs [108], with the largest and oldest being Muhammadiyah, PERSIS, and Nahdlatul Ulama [109]. Nahdlatul Ulama has an estimated 91.2 million members, while Muhammadiyah has around 60 million [110]. With such large memberships, both Muhammadiyah and Nahdlatul Ulama actively engage in family planning programs.

Grounded in the principle of ensuring that children grow up healthy, nurtured in faith, and receive complete parental affection, Muhammadiyah emphasizes that birth spacing is not in conflict with Sharia. Instead, it is regarded as a moral and spiritual responsibility aligned with the commands of Allah SWT. This position is formally articulated in a fatwa issued by the *Muhammadiyah Majelis Tarjih* [102]. Meanwhile, Nahdlatul Ulama (NU) has also endorsed family planning. As early as its first conference in April 1960, NU addressed the issue. At its 28th conference in November 1989, it formally permitted the use of intrauterine devices (IUDs), equating them with *‘azl* (coitus interruptus) and other contraceptive methods [111]. Beyond Islamic CSOs, the Indonesian Confucian Religious Council (*Majelis Agama Konghucu Indonesia*) and the Communion of Churches in Indonesia (*Persekutuan Gereja Indonesia*) also do not prohibit family planning [112].

Although their guidelines generally apply to married couples, without distinguishing whether they married during adolescence, these documents and discussions provide a framework for offering family planning services to married adolescents. For unmarried adolescents, the emphasis is placed on moral education and the prevention of premarital sex. In addition to issuing documents and conducting discussions, religious leaders in Indonesia, through the Interfaith Forum for Family Welfare and Population (*Forum Antar Umat Beragama Peduli Keluarga Sejahtera dan Kependudukan*, abbreviated as Fapsedu), play an active role in supporting the national family planning program and the Generation Planning Program (*Generasi Berencana,* abbreviated as GenRe). They do so by promoting adolescents’ knowledge, attitudes, and behaviors related to reproductive health and preparation for family life. Fapsedu also contributes to raising awareness to prevent the “Triad of Adolescent Reproductive Health” problems: premarital sex, early marriage, and substance abuse [113]. The promotion and discussion of such healthy behavior has also been performed by other CSOs such as Naysiatul Aisyiyah, Fatayat NU, Ikatan Pelajar Muhamamadiyah, and the Information Center and Movement for the Prevention of Early Marriage *(Sentra Informasi dan Gerakan Antisipasi Pernikahan Dini*, abbreviated as *SIGAP DINI*) [114–117].

#### E) Tailoring program design to diverse contexts

Programs are contextually adapted to address variations in cultural, religious, and socioeconomic environments, thereby enhancing their relevance and sustainability. In Indonesia, CSOs respect Sharia law by limiting contraception services to married adolescents. Offering contraception to adolescents appears to be a challenging task, as it is viewed as controversial by society due to moral and religious concerns, conflicts with social and cultural norms, and growing apprehensions regarding adolescents’ sexuality. By respecting local norms and integrating community values into program delivery, CSOs in Indonesia are tackling these barriers. In Kenya, CSOs tailor messages based on tribal, religious, and political influences. In this country, CSOs have strategically tailored AFP messages by taking into account tribal, religious, and political influences. In pastoralist communities such as Mandera, Samburu, and Wajir, where cultural norms favor large families and traditional gender roles, CSOs work closely with local leaders, elders, and religious figures to shape messages that align with community values [118]. Programs like Delivering Sustainable and Equitable Increases in Family Planning (DESIP) engage community and faith leaders to promote family planning as a way to protect maternal and child health, using local languages and culturally relevant narratives [119]. These tailored approaches help reduce resistance and foster trust among adolescents and their families. Additionally, CSOs collaborate with county-level political leaders to integrate family planning into local health policies, increasing support for adolescent reproductive health initiatives. By aligning their efforts with tribal customs, religious teachings, and political priorities, CSOs in Kenya have enhanced adolescent access to and acceptance of modern contraceptive methods [120].

In Cameroon, messaging acknowledges family size preferences and economic considerations tied to culture. Cameroonian communities often hold cultural beliefs that favor large families, seeing many children as financial security and a safeguard against high child mortality, which creates resistance to birth-limiting messages. However, the BornFyne PNMS family planning initiative has successfully countered such barriers with a tailored, culturally-conscious approach. By delivering educational audio content in local dialects, the program directly responds to concerns about contraceptive side effects, incorporates men as active participants, and aligns messaging with religious sensitivities—all of which have enhanced local acceptability [75].

The summary of the CSOs role in cultural and religious adaptation for sustaining family planning programs is available in Table 3.

**Table 3:** The summary of CSOs’ role in cultural and religious adaptation for sustaining family planning programs

Thematic Role	Country Example	Key CSO Activities
Integrating Local Cultural and Religious Values	Bangladesh, Cameroon Nigeria	BRAC, Marie Stopes, community gatekeepers and culturally approach, reframing FP in child spacing, and CCREAD.
Engaging Community Gatekeepers and Families	Bangladesh, Cameroon, India, Kenya	Save the children, SNEHA, CCREAD.
Utilizing Innovative and Interactive Approaches	Bangladesh, Madagascar	Educate community through theatre, storytelling, and games; Pathfinder International’s programs, raise awareness to counter stigma and myths.
Addressing Sociocultural Barriers through Dialogue and Transformation	India, Indonesia, Malawi	Gender transformative approaches, intergenerational dialogue; collaborate with family, community and health workers; issued FP regulations and recommendations framing with religious values; discussions among religious leaders; raising awareness to reproductive health and preparation for family life, prevent adolescents pregnancy and early marriage
Tailoring Program Design to Diverse Contexts	Cameroon, Indonesia, Kenya	Respect on religious law, political influences, DESIP program, BornFyne PNMS, educational audio content in local dialects

## Discussion

The findings of this study on AFP reveal that across nine Asian and African countries, adolescent pregnancy remains a significant challenge, driven by sociocultural, economic, and policy barriers. In Indonesia, for instance, 60% of adolescents aged 16–17 engage in premarital sex, contributing to high rural birth rates (51/1,000).^105^ In Myanmar, the ongoing political crisis has further exacerbated gaps in reproductive health access, with 16% of girls married before the age of eighteen [19, 121]. India’s RKSK program utilizes peer education and digital outreach to reach its 253 million adolescents [21], while Bangladesh’s National Adolescents Health Strategy (2017–2030) is designed to empower youth [24]. In Africa, Malawi’s policies are progressive [29–36] but face slow implementation. In Nigeria, 23% of girls aged 15–19 are already childbearing^,42^ and Kenya’s adolescent pregnancy rates remain high among those with low incomes, with 21% of 15–19-year-old women in the lowest wealth quintile pregnant compared to 8% in the highest [37], Madagascar and Cameroon experience early sexual debut (ages 15–16) and rising rates of sexually transmitted infections (STIs) [50, 122], despite cultural stigma surrounding contraception. These findings illustrate how disparities in rural access, limited funding, and sociocultural resistance continue to impede progress, even where national programs and policies exist, thereby highlighting the need for focused and sustainable interventions.

This study demonstrates that CSOs play a pivotal role in preventing early sexual debut and sustaining AFP programs by fostering community engagement and participation. Community education and active engagement are crucial in shaping and guiding family planning initiatives [123]. Evidence shows that community-based education and mentoring strategies significantly enhance both awareness of and active participation in family planning programs [124], mainly for those who are married in adolescent age. Across diverse contexts, CSOs promote peer-led education and youth empowerment, enabling adolescents to access accurate and relatable sexual and reproductive health (SRH) information while also becoming active advocates in their communities. They build trust and local ownership by collaborating with parents, teachers, religious leaders, and local authorities, ensuring that services are culturally aligned and stigma is reduced. Many CSOs combine digital tools with grassroots approaches, as illustrated by Myanmar’s Baykin 2 app and Cameroon’s BornFyne initiative, which extend the reach of programs and reflect local input. Programs in Indonesia, Nigeria, and Kenya further demonstrate that contextual and cultural adaptation is essential, with messages framed in ways that resonate with community values. Beyond service delivery, CSOs also strengthen advocacy and social accountability by ensuring that youth voices inform policies and that interventions reflect actual needs. For example, in India, civil society-led peer education programs under the National Adolescents’ Health Program have significantly improved knowledge and attitudes toward SRH [69]. These approaches are especially critical in conflict-affected regions such as Myanmar, where civil society provides essential family planning support to vulnerable adolescents despite systemic disruptions. Collectively, these strategies demonstrate that sustaining AFP programs depends on empowering adolescents and embedding initiatives within culturally sensitive, community-driven frameworks.

Regarding partnerships and collaboration, CSOs contribute to sustaining AFP programs by building strong relationships with governments, international agencies, and community stakeholders. Evidence shows that CSOs actively contribute to policy advocacy by joining technical working groups, shaping national strategies such as FP2030 in Nigeria and RMNCH+A in India, and ensuring that youth needs are reflected in planning and accountability platforms like Track20. Joint implementation initiatives—such as Indonesia’s *Posyandu* services and BRACs culturally grounded programs in Bangladesh—illustrate how CSOs bridge service gaps and build local trust. In countries like Madagascar and Cameroon, partnerships with agencies such as UNFPA and FID expand outreach and address gender based barriers. Cross-sector alliances with youth-led groups, traditional leaders, and private sector actors, as seen in Kenya and Nigeria, further enhance scale and sustainability. These findings support the argument that FP2030’s success relies on strong, enduring partnerships between governments and CSOs, empowering civil society to drive family planning efforts and ensuring government accountability. Strong civil society engagement accelerates progress toward FP2030 targets and the Sustainable Development Goals [125]. Importantly, CSOs also strengthen accountability through data-driven planning, providing insights that improve transparency and inform evidence-based decisions [126]. Taken together, these collaborations ensure that AFP programs are inclusive, locally owned, and responsive to the complex realities of adolescents’ sexual and reproductive health, while also enhancing program sustainability and broader accessibility.

CSOs also play a crucial role in embedding family planning interventions within cultural and religious contexts to foster acceptance and trust. Religious traditions and gender dynamics significantly influence the uptake of family planning, as differing interpretations of these traditions affect the perceived acceptability of family planning [127]. In Bangladesh, Nigeria, and Indonesia, family planning messages are reframed around locally valued concepts such as child spacing, parental responsibility, and faith-based duties, enabling CSOs to engage effectively with religious leaders, parents, and traditional gatekeepers. Programs in Cameroon and Kenya further demonstrate that adapting language, involving male champions, and respecting local norms can reduce stigma and open dialogue around contraception. Interactive strategies, such as theater, storytelling, and peer-led workshops, help adolescents and families engage with sensitive topics in culturally meaningful ways, while intergenerational and gender-transformative dialogues address deeply rooted taboos. Although many religious leaders understand the importance of family planning, they remain hesitant to promote modern contraceptive methods [128]. Engaging and mobilizing them is therefore crucial to advancing family planning efforts and supporting progress toward the Sustainable Development Goals. In contexts with strict religious frameworks, such as Indonesia, CSOs align services with Sharia principles by focusing on married adolescents and promoting sexual abstinence for unmarried adolescents, ensuring compliance with local laws while continuing to promote reproductive health. These adaptive approaches demonstrate that when CSOs respect community values and collaborate with gatekeepers, family planning programs become more relevant, ownable, and sustainable. In addition, since sociodemographic characteristics affect contraception usage, family planning efforts should target education disparities, encourage earlier family planning, and extend services for low-income women while also reaching wealthy urban women [129]. Effective empowerment programs for sustainability depend on a deep understanding of local contexts and the capacity to tailor strategies to the specific needs of each community [130].

This study shows how supportive, participatory, and context-sensitive approaches can help CSOs do their jobs better, which can help keep family planning programs going. This information is beneficial for professionals working in adolescent family education and reproductive health programs. The results inform how to incorporate community involvement, partnerships, and cultural adaptation into program design. Furthermore, it offers health professionals, program managers, and policymakers ideas on how to make services more accessible, acceptable, and sustainable. Scientifically, this study advances understanding in adolescent and reproductive health by evidencing how CSOs can bridge service delivery gaps, foster trust, and ensure equitable access to contraception for those most in need.

The study provides valuable information from multiple nations; nevertheless, it has several limitations. For example, it relies on data from CSOs, which could introduce self-reporting bias, and the maturity of the programs varies from place to place, making it difficult to compare. The qualitative design helps us understand the context better, but it makes it more challenging to generalise about how well the intervention worked. Future research should incorporate mixed-methods approaches, including quantitative impact evaluations, to strengthen evidence on program outcomes. Longitudinal studies that track the sustainability of interventions over time, particularly in conflict-affected settings, would further enhance our understanding. Standardized metrics to evaluate cultural adaptation tactics in various contexts are also required, as is a more thorough investigation of the intersectional issues (such as gender and poverty) that exacerbate access hurdles.

## Conclusion

This study highlights the critical role of civil society in advancing sustainable AFP programs across nine Asian and African countries despite sociocultural, political, and resource challenges. Three key pillars emerged as foundational to program success. CSo’a Engagement and participation, partnerships and collaboration, and cultural and religious adaptation is essential for sustaining AFP programs. Governments, partners, and donors should strengthen CSOs to sustain and enhance the impact of AFP programs. Some actions can be taken, such as through targeted investments in youth empowerment, local ownership, grassroots mobilization, and gatekeeper involvement in activities. The combination of digital innovations with community-based models, as well as the application of culturally sensitive designs, may improve reach and relevance in the AFP program. Partnerships should be deepened through government and CSO alliances, which might help support partnerships. Programs such as joint implementation, cross-sector collaboration, and data-driven accountability might enhance the benefits. Moreover, by integrating local cultural and religious values, engaging families and leaders, and utilizing interactive, dialogue-based approaches, we can help address sociocultural barriers. Lastly, by institutionalizing these strategies in health systems, it might ensure lasting acceptance, effectiveness, and community ownership.

### Impact of the study

This study highlighted the essential role of CSOs in enhancing reproductive health services in diverse settings, especially in supporting family education programs. CSOs minimize barriers to family planning education and care, especially in conflict-affected and faith-sensitive countries, by increasing community engagement, fostering government partnerships, and adapting to cultural and religious contexts. Collaboration among partners and focusing on adolescents’ needs can enhance maternal and reproductive health outcomes more equitably and sustainably, lowering unplanned births and health inequities.

## Data Availability

Not applicable.

## Abbreviations

AYSRH: Adolescents and Youth Sexual and Reproductive Health
BRAC: Bangladesh Rural Advancement Committee
CCREAD: Centre for Community Regeneration and Development
CSOs: Civil Society Organizations
HIV&AIDS: Human Immunodeficiency Virus & Acquired Immune Deficiency Syndrome
LKD: *Lembaga Kemasyarakatan Desa*
LMICs: Low- and Middle-Income Countries
MOHFW: Ministry of Health and Family Welfare
NDHS: Nigeria Demographic Health Survey
PASHMINA: *Pelayanan Remaja Sehat Milik Nasyiatul Aisyiyah*
PPROM: Premature Rupture of the Membrane
*RKSK*: *The Rashtriya Kishor Swasthya Karyakram*
SNEHA: Society for Nutrition, Education & Health Action
STI: Sexually Transmitted Infection
UNFPA: United Nations Population Fund
UNICEF: United Nations Children Fund
WHO: World Health Organization
